# Supplementary material for: CRISPR/Cas12a-Based One-Tube RT-RAA Assay for PoRV Genotyping
Source: Int J Mol Sci. 2025 Jul 16;26(14):6846. doi: 10.3390/ijms26146846 (PMC12295710; doi:10.3390/ijms26146846)
Supplement: Supplementary file 1 [file ijms-26-06846-s001.zip › ijms-3708899-supplementary.pdf]

Supplementary Figures legends

Supplementary Figure S1. Purification of CRISPR/LbCas12a protein

The red boxed line in the figure shows the location of the target protein.

Supplementary Figure S2. Multiple Sequence Alignment of PoRV Gene Fragments

The sequence positions of the designed crRNA are indicated by black boxs.

Supplementary Figure S3. Optimization of RT-RAA reactions

(a)Screening of optimal primers for RT-RAA. (b) Optimizing the optimal reaction time for RT-RAA. (c) Optimization of primer concentration for RT-RAA. (d) Exploring the optimal reaction temperature for RT-RAA.

Supplementary Figure S4. Validation of the feasibility of the RT-RAA-CRISPR/Cas12a assay.

To validate the feasibility of RT-RAA-CRISPR/Cas12a method for detecting G4, G5 and G9, the results were displayed by multifunctional enzyme labeling apparatus and fluorescent tubules.

Supplementary Figure S5. Detection of clinical samples

G4, G5, and G9 infections were detected in 31 clinical samples from pigs using qPCR assay, and the results were shown in the form.

Supplementary Figure S1. Bi et al.

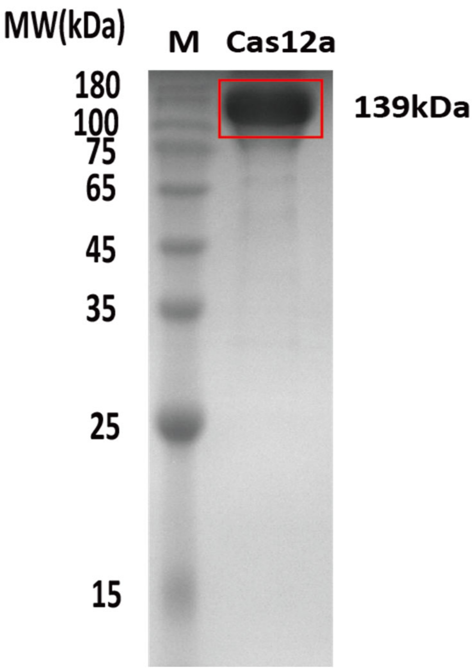

Supplementary Figure S2. Bi et al.

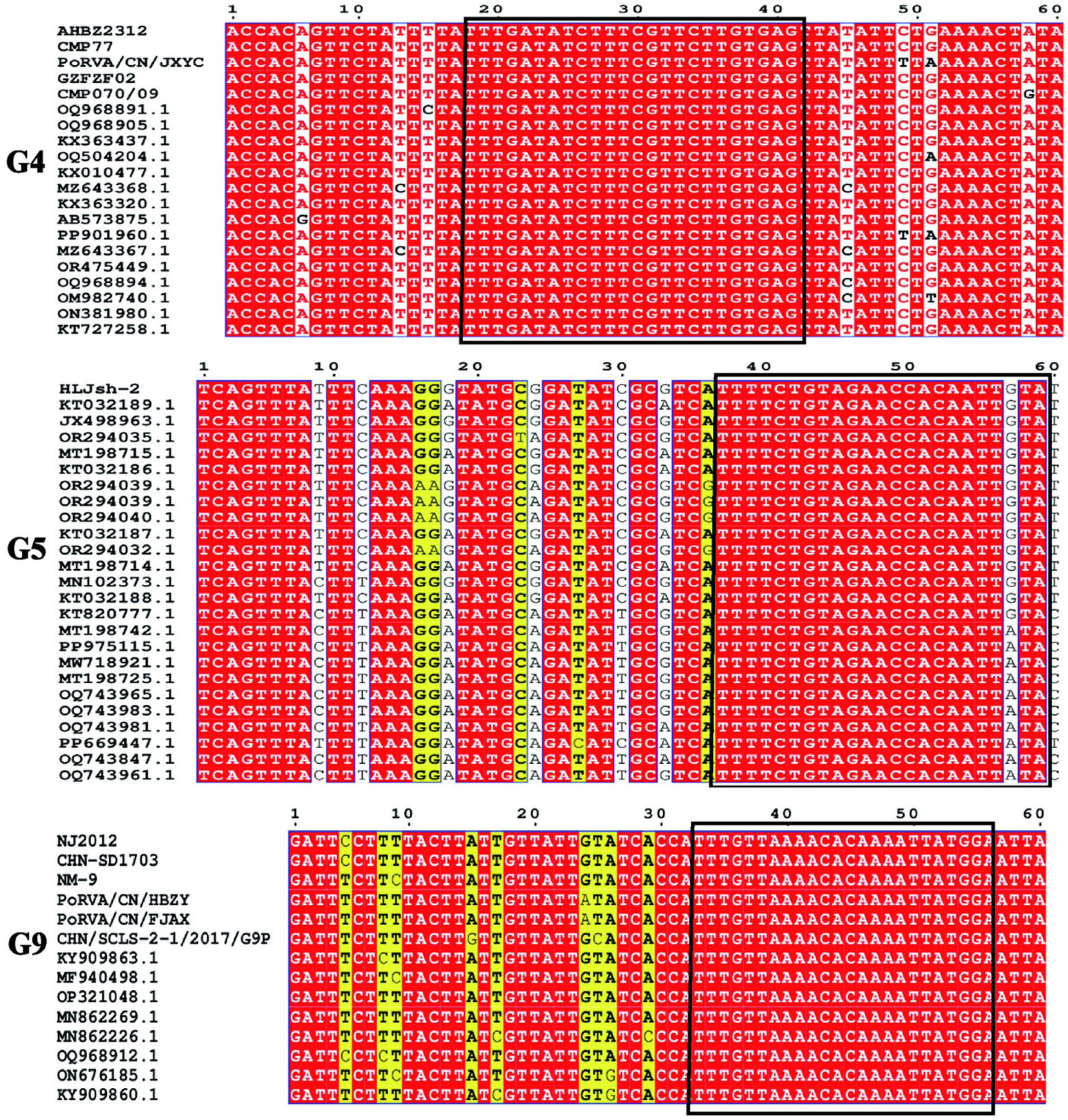

Supplementary Figure S3. Bi et al.

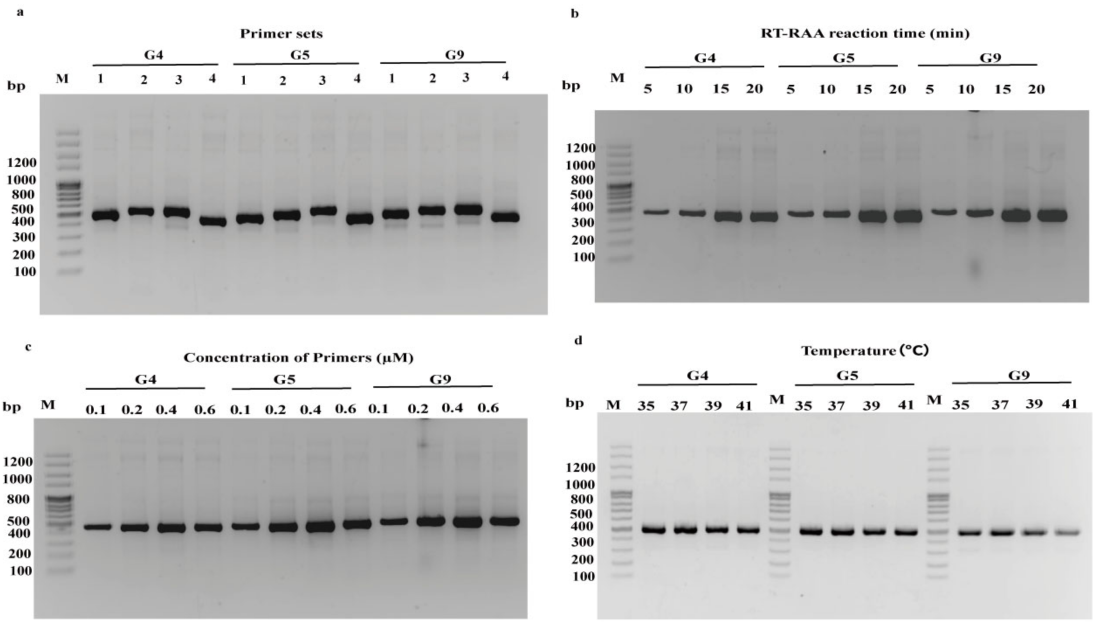

Supplementary Figure S4. Bi et al.

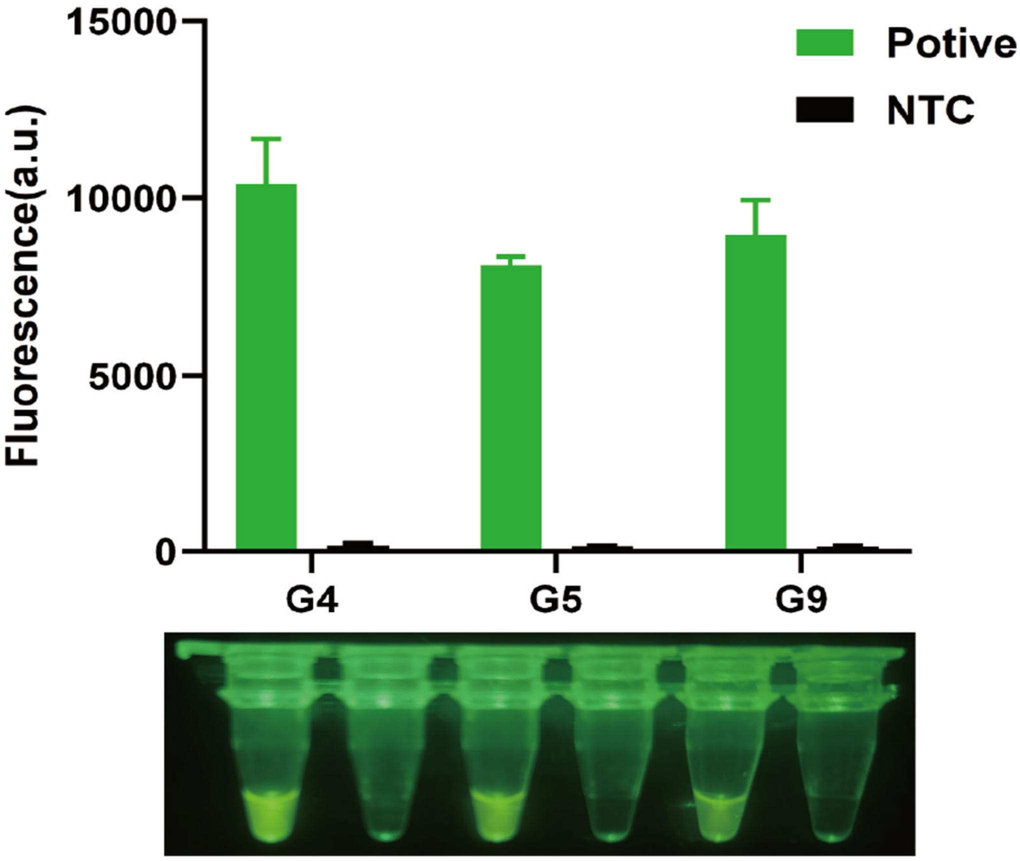

Supplementary Figure S5. Bi et al.

G4

	1	2	3	4	5	6	7	8
I	-	+	-	-	-	-	+	-
II	-	-	-	-	+	-	-	-
III	-	-	+	+	-	-	-	-
IV	-	+	-	-	-	-	+	-

G5

	1	2	3	4	5	6	7	8
I	-	-	-	-	-	-	+	-
II	-	-	-	-	-	-	-	-
III	-	-	-	-	-	-	-	-
IV	-	-	-	-	-	-	-	-

G9

	1	2	3	4	5	6	7	8
I	-	+	+	-	+	-	+	+
II	+	+	-	-	+	-	-	-
III	-	+	-	-	+	+	+	+
IV	-	+	-	-	-	-	+	-
